# RETRACTION: Shear‐Induced ITGB4 Promotes Endothelial Cell Inflammation and Atherosclerosis

**DOI:** 10.1155/omcl/9828461

**Published:** 2026-08-04

**Authors:** Oxidative Medicine and Cellular Longevity

RETRACTION: X. Kong, S. Chen, S. Luo, et al., “Shear‐Induced ITGB4 Promotes Endothelial Cell Inflammation and Atherosclerosis,” *Oxidative Medicine and Cellular Longevity* 2022, no. 1 (2022): 1–12, https://doi.org/10.1155/2022/5842677.

The above article, published online on 25 October 2022 in Wiley Online Library (wileyonlinelibrary.com), has been retracted by agreement between the Journal Editor, Jeannette Vasquez‐Vivar; and John Wiley & Sons Ltd.

The retraction has been agreed upon following an investigation into image concerns raised on PubPeer [1]. Similarities were identified between the β‐actin control bands presented in Figures 2b and 5e, which are associated with different experimental groups. The authors were unable to provide verified original images or a sufficient response to the concerns raised. Therefore, it is agreed that the article must be retracted.

The authors disagree with the retraction.
